# Fused Deposition Modeling of Polymer Composites: Development, Properties and Applications

**DOI:** 10.3390/polym17081054

**Published:** 2025-04-14

**Authors:** Aldobenedetto Zotti, Teresa Paduano, Francesco Napolitano, Simona Zuppolini, Mauro Zarrelli, Anna Borriello

**Affiliations:** Institute for Polymers, Composites and Biomaterials, National Research Council of Italy, P.le Fermi, 1, 80055 Portici, NA, Italy; aldobenedetto.zotti@cnr.it (A.Z.); teresapaduano@cnr.it (T.P.); francesconapolitano@cnr.it (F.N.); simona.zuppolini@cnr.it (S.Z.); anna.borriello@cnr.it (A.B.)

**Keywords:** fused deposition modeling (FDM), fused filament fabrication, polymer matrix composites (PMCs), thermoplastic polymers, 3D printing

## Abstract

This review summarizes recent research advancements in thermoplastic composites used in Fused Deposition Modeling (FDM) processes. Since its development in 1988, FDM has emerged as one of the primary emerging technologies of Industry 4.0, receiving attention in fields such as industrial manufacturing, automotive, aerospace, and others, particularly for rapid prototyping and customization. The intention is to make available a guideline for 3D printing researchers, analyzing the properties and performance characteristics of different polymers and polymeric composites. The review analysis covers various reinforcing agents, including particles/nanoparticles, short fibers, and long fibers, identifying critical parameters of the FDM process which affect printed part quality, integrity and final geometry. Major attention is devoted to the different techniques employed for composite filament fabrication, mostly for structural elements and parts. An extensive overview of various FDM composites and fiber-reinforced composites by polymer matrices such as PLA, ABS, and PEEK is presented, with their mechanical and thermal properties reported for specific applications. Current challenges and prospective future research directions are also outlined, mainly focusing on the enhancement of material performance and sustainability.

## 1. Introduction

**Figure 1:**
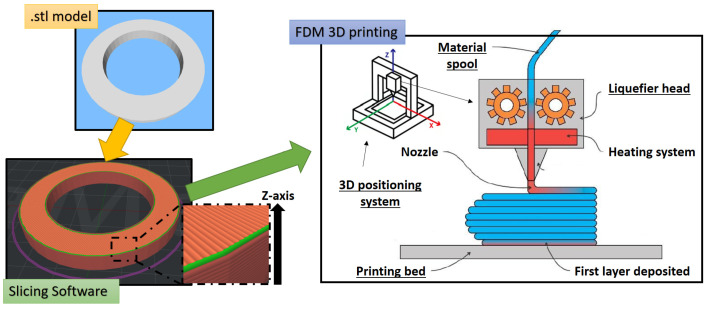
Schematic illustration of the working principle for FDM process.

The term additive manufacturing (AM), commonly known as three-dimensional (3D) printing, refers to a group of techniques by which complex 3D objects are built through the progressive layer-by-layer deposition of material. AM includes several polymer manufacturing techniques such as Selective Laser Sintering (SLS), Inkjet Printing (IP), Stereolithography, Fused Deposition Modeling (FDM), and others [1,2]. Among these 3D printing processes, the FDM method, also known as Fused Filament Fabrication (FFF), is the most employed technique due to its simplicity and versatility. Since its development in the late 1980s by S. Scott Crump and subsequent commercialization in 1990 by Stratasys [3], FDM has gained more and more attention from an industrial and research point of view, until it became one of the leading emerging technologies of Industry 4.0 [4]. In the FDM process, a filament of the selected material is pushed, by counter-rotating gears, into a heated nozzle of precise diameter and deposited layer-by-layer in a semi-solid state on the previously built layer. Figure 1 shows the structure of a standard FDM 3D printer, highlighting its main parts, i.e., the printing bed, the liquefier head, the 3D positioning system, and the material spool.

The first step in building an FDM object involves the realization of the object model’s .stl formatted file using a computer-aided design (CAD) software (Onshape CAD Software). The .stl file is then imported into a slicing software, where the model is divided into thin two-dimensional layers along the z direction. The 3D positioning system controls the printing head movements, moving along the two-dimensional contour information provided by the sliced model to produce the first layer in the x-y plane. Subsequently, the printing bed shifts in the z direction, moving away from the liquefier head, enabling it to print the subsequent layer on the previous one. The process continues until the sliced model is completely printed. Upon removal of the built object, only the supports still need to be removed, resulting in limited waste production compared to traditional subtractive manufacturing processes [5].

Compared to traditional manufacturing techniques, FDM is characterized by different advantages, such as the following [6,7]:

- ability to print complex geometries;
- high printing speed without the need for post-production processes to refine the manufacts;
- no chemical changes in the raw material during the printing process, preserving the virgin material properties;
- limited maintenance required and safe system due to the simple construction principle;
- less material waste.

Furthermore, over the last decades, an increasing number of thermoplastic polymers have become accessible for use in FDM, such as acrylonitrile butadiene styrene (ABS), polyether-ether-ketone (PEEK), polypropylene (PP), polyactide acid (PLA), and so on [8]. However, the process is characterized by different constraints that limit its applicability in advanced applications. For instance, FDM-manufactured products generally exhibit inferior mechanical performance than their traditionally manufactured counterparts, and this weakness can be attributable to either the poor strength at the interface between two adjacent printed filaments or that of the filament itself [9]. Shrinkage and anisotropy of the printed products are additional drawbacks of the FDM technique related to both the polymeric nature of the printed material as well as to the printing process [7]. In an effort to expand FDM for advanced materials production (aeronautical and aerospace, medicine, energy, etc.) different strategies have been proposed. However, the most efficient and cost-effective approach involves the addition of functional fillers to the polymer matrix. Among numerous micro- and nanomaterials, carbon nanotubes (CNT), graphene nanoplatelets (GNP), short fibers, and metal and oxide nanoparticles have attracted considerable interest due to their distinctive electrical, thermal, and mechanical properties. Filler-loaded 3D-printable thermoplastic polymers enable not only enhanced mechanical and thermal properties, as well as wear resistance, but also induce new functional properties such as electrical conductivity and antimicrobial properties. Fillers can also improve the processing capability of the polymer filaments: the polymer extrusion stability is significantly influenced by fillers addition, reducing the shrinkage and improving the surface roughness of the FDM product, with a consequent reduction in post-processing time and thus costs [10,11].

**Figure 2:**
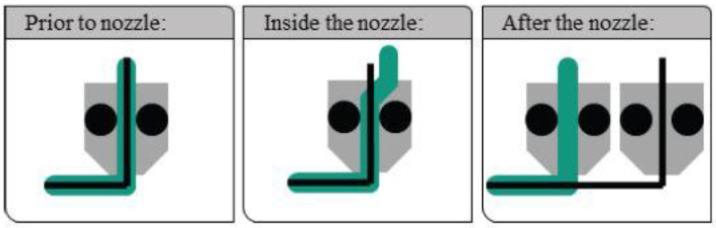
Schematic illustration of FDM process used for CFRC printing (in cyan the polymer, in black the carbon fibers) [12].

Novel technologies have enabled the manufacturing of continuous fiber-reinforced composites (CFRC) using modified FDM processes. There are three different methods to incorporate continuous fiber within a matrix, as schematically illustrated in Figure 2 [12], with major differences in terms of timing and location of fiber/matrix mixing.

In the first method, identified as “prior to nozzle” since the fiber/matrix mixing occurs before the filament enters in the extrusion head, prefabricated composite filaments are utilized to print the part, and conventional FDM apparatus can be employed. In the second method, indicated as “inside the nozzle” since continuous fibers are impregnated directly in the printing head, the thermoplastic resin and the reinforcement are supplied by two separate rollers and the impregnation is performed in a heated nozzle, capable of printing both fiber and matrix in a single step. The printing setup is complex and one of the primary challenges consists of avoiding air inclusions likely to promote debonding [13]. The third approach is identified as “after the nozzle” since one printing head prints the polymer matrix with a concentric or isotropic pattern, while a second head prints the continuous fibers on the previously deposited polymer matrix layer. In this way, it is possible to gain a larger control of the fiber volume fraction; anyway, inappropriate fiber deposition could induce defects in the 3D-printed composites, but temperature-controlled post-processing can significantly increase the strength [14]. Moreover, the continuous fibers can be sized with a polymer that increases the stickiness toward the underlying layer [15]. The latter two methods, compared to the “prior to nozzle” approach, are more susceptible of worse surface impregnation due to the lack of quality control upon impregnation but, at the same time, allow for greater customizability. The implementation of the “after the nozzle” method led to the possibility to print reinforcements in specific areas only, optimizing the element stiffness profile according to the stress analysis.

Although reinforcing 3D printing thermoplastic polymers with fillers and/or continuous fibers is a promising field of research, there are still some challenges that must be resolved in FDM composite materials.

First of all, the systems must be able to be processed in feedstock filaments of precise diameter (i.e., 1.75 or 2.85 mm) and have a uniform cross-section. This could be very difficult to achieve, especially in the case of nanocomposites or CFRC, and therefore a good-quality manufact always requires a comprehensive characterization in terms of melt viscosity, strength, modulus, and ductility. The melt flowability and the viscosity of the filament are strongly affected by the filler (particles or fibers) content; moreover, during the printing, fillers can cause nozzle clogging due to particles clustering and void formations [16]. If CFRP are not properly designed and processed, delamination due to poor fiber/matrix adhesion can occur, strongly affecting the final material strength. For these reasons, FDM of composite materials is a very challenging research field, whose study is motivated by the prospective of developing high-performance materials using a fast, cost-effective, and flexible technique.

This review summarizes very recent research advancements in thermoplastic composites used in FDM processes, considering particles/nanoparticles, short fibers, and long fibers as reinforcing agents. The critical parameters to consider in FDM processes and the different techniques to produce nanocomposite feedstock filaments are presented, analyzed, and discussed. The main section of this review describes in detail the properties and applications of nanocomposites prepared for FDM, listed on the basis of the polymer matrix (PLA, ABS, etc.). In a nutshell, this review aims to provide a guideline for researchers working in the field of 3D printing to understand the various polymer and polymeric composites’ properties and performance.

## 2. FDM Processes on Polymer and Polymer-Based Composites

### 2.1. Parameters Affecting FDM Processes

**Figure 3:**
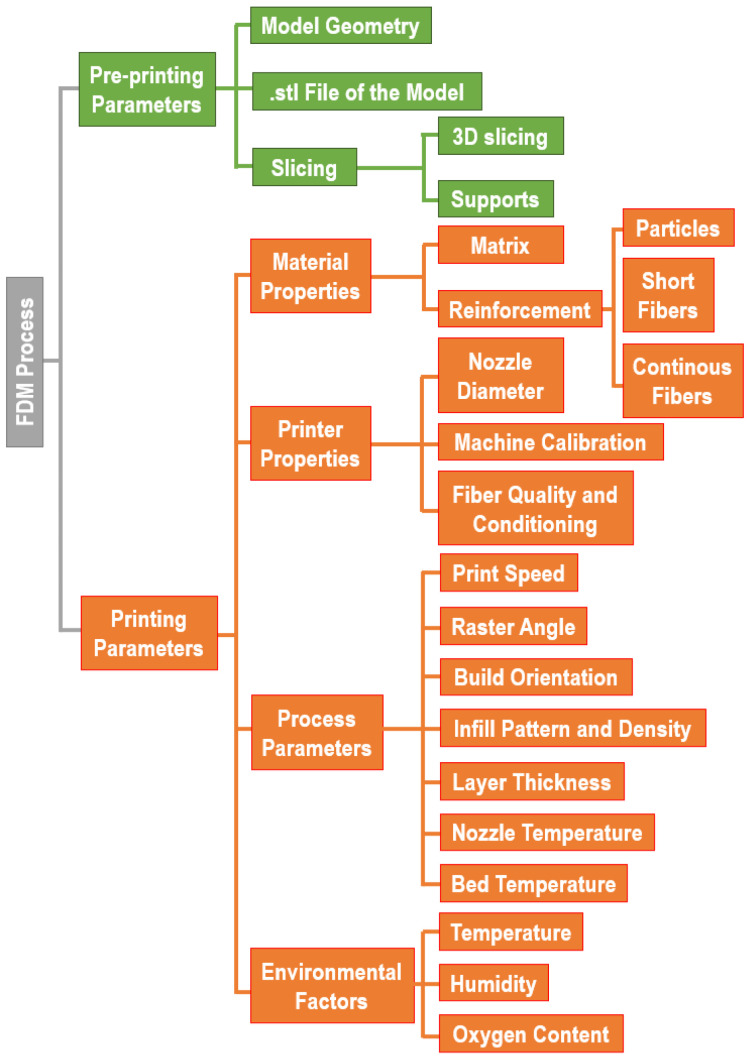
List of pre-printing and printing parameters affecting FDM process.

Numerous factors can significantly affect the properties (both physical and esthetic) of FDM products; in 2018, Banjanin et al. [17] identified and classified the main parameters affecting the mechanical properties of 3D-printed composites in FDM process. Figure 3 reviews these parameters, grouped into two macro classes, i.e., “pre-printing” and “printing” parameters.

#### 2.1.1. Pre-Printing Parameters

As previously stated, to print an object with FDM (or with general AM) technology, it is necessary to have a model created using CAD programs and exported in specific formats. The most common format used to represent the object model is STL, but nowadays many other formats are available, such as OBJ, AMF, and 3MF [18], and their choice depends on the user’s needs. STL is the universally accepted format, OBJ is preferred for multi-color and high-precision systems (2 or more printing heads), and formats like AMF and 3MF are used in modern 3D printers for complex shape manufacts requiring high resolution. The slicing software is responsible for slicing the manufact in 2D slices along the z-axis (namely the axis perpendicular to the printing plane) and providing the spatial sequence in which these slices must be printed. Moreover, this software allows for the creation of the so-called “supports” needed to build complex geometries, as well as defining the typology of the “platform” to be implemented (skirt, brim, or raft), establishing a smooth filament flow in the early stages of printing and improving the adhesion of the part onto the printing bed [19].

#### 2.1.2. Printing Parameters

The printing parameters are the main factors to set and optimize with respect to the performance and architecture of the printed composites. These parameters define the material properties, and any slight changes to the machine or material parameters will significantly affect the properties and quality of the final printed product [20,21].

The material properties, in terms of the polymer matrix and reinforcement, are the main factors affecting the printed part properties. Thermoplastic polymers with an amorphous or semi-crystalline nature are generally preferred: in fact, polymers with high crystallinity are characterized by a high shrinkage level and solidify slowly, inducing an inhomogeneous deposition leading to internal stresses or final part distortions. Conversely, amorphous polymers solidify faster, showing less shrinkage percentage and dimensional variations [12]. In the case of composites, the reinforcement features (such as shape, dimension, and chemical nature) play a crucial role in the final manufact’s performance. In addition, the filler dispersion technique within the matrix can remarkably affect the final product integrity and quality [22]. The 3D feeding material is commonly loaded in the printer in the form of a filament with a diameter of 1.75 mm or 2.85 mm and a tolerance of ±0.05 mm [23]. It is noteworthy that filament conditioning is crucial to prevent moisture uptake and polymer degradation, and thus filament dehumidification is necessary, especially for hydrophilic polymers such as polyvynil alcohol (PVA).

One of the most influential parameters in 3D printing is the nozzle temperature; indeed, higher temperatures promote interlamellar bonding and may reduce voids during the printing stage, thereby improving mechanical properties of the printed parts. Conversely, high temperatures can excessively reduce viscosity, leading to an excess of feeding material and thus detrimentally affecting the mechanical properties. This was reported by Rivera-López et al. [24], investigating the effect of nozzle temperature, ranging between 180 and 260 °C, on the tensile and flexural properties of FDM PLA-based parts. These conclusions are supported by the work of Farashi et al. [25], which reviewed, using a meta-analysis study, the effect of nozzle temperature on the tensile strength of FDM-printed parts; in the same study, the authors report an inverse proportional relationship between printing speed and tensile strength. Nozzle diameter also influences the properties of printed materials. A smaller diameter results in a higher resolution of the manufact, but clogging of the composite filaments is likely to occur if the nozzle is too narrow [26] (the standard nozzle diameter for a FDM printer is generally 0.4 mm [27]). A lower layer thickness increases the packing of the deposited polymer, promoting adhesion between the raster fibers and consequently increasing the mechanical properties; moreover, the surface resolution remarkably increases, reducing the layer thickness [28].

**Figure 4:**
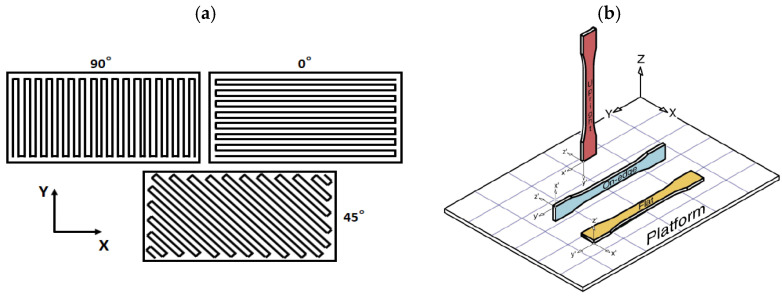
(**a**) Raster angles and (**b**) printing orientations in FDM.

Raster angle represents the angle of deposited raster relative to the X axis (see Figure 4a) and it has a remarkable effect on the mechanical properties of the printed part [29]. A 0° raster angle leads to the highest ultimate tensile strength, with a fracture perpendicular to the applied load due to the raster failure. Increasing the raster angle up to 90° dramatically reduces the ultimate strength as failure occurs at the interface among the rasters. The fracture path of the ±45° exhibits both failure patterns, along with the highest toughness and ductility. The reason for the ductile behavior of the ±45° raster is attributable to the changes in the mesostructural layout that occur due to the progressive damage of the interphase among the rasters; in a nutshell, the raster fibers, which are not oriented parallel or perpendicular to the loading direction, undergo an alignment toward the loading direction, considerably stretching before breaking [30]. The raster angle is not the only parameter linked to the printing orientation but, as reported in Figure 4b, three different “building” orientations can be identified, i.e., through-the-thickness (flat), through-the width (on-edge), and through-the-length (upright). These building orientations play a relevant role in the mechanical properties and built time for FDM-printed parts. Eryıldız et al. [31] observed a 36% reduction in tensile strength for upright-printed samples compared to the flat-printed ones. Moreover, the printing time increases from 29 min to 231 min, respectively, from the flat- to the upright-printed sample.

Infill density represents the amount of material used to fill the printed part volume; optimizing this parameter, it is possible to tune the part filling degree with a consequent variation in density and strength. Infill density is generally expressed as a percentage, with a 100% density value corresponding to a full solid printed object.

The infill pattern indicates the filling geometry and defines the moving action of the printing head within the X-Y plane during material deposition. Analogously to the infill density, the pattern is also likely to influence the printing time, the density, and the final mechanical properties [32].

Environmental factors (temperature, humidity, and oxygen content) influence the viscosity, surface tension, and solidification rate of the deposited polymer and can therefore affect the achievable printing performance [33].

### 2.2. Composite FDM Filament Fabrication

**Figure 5:**
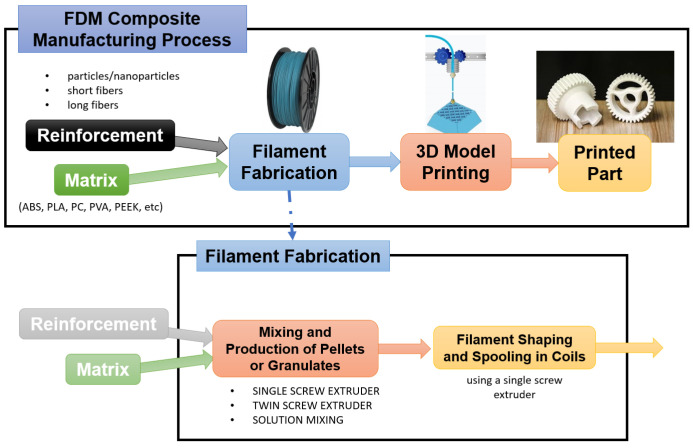
Schematic illustration of FDM printing of a composite material.

The feedstock filament is the primary material employed in FDM printing, and for this reason it is crucial to consider and fully comprehend its manufacturing process. Filament can comprise both pure polymer (for example ABS, PLA, PC, etc., [8]) and composite material, consisting of a pure polymer as a matrix and a reinforcing phase (short, micro-, and nano-particles or long fibers). Filament fabrication is a critical step in composite materials FDM processes; as reported in Figure 5, the fabrication of a composite filament can be divided into two main steps, i.e., (1) the composite mixing and pellet fabrication, and (2) the filament shaping and spooling [5].

#### 2.2.1. Composite Mixing and Pellets Fabrication

The initial step in composite filament fabrication involves the mixing of a base polymer and a reinforcement. The main mixing techniques employed in composite filament fabrication for FDM processes are melt mixing (utilizing single- or twin-screw extruders) and solution mixing.

**Figure 6:**
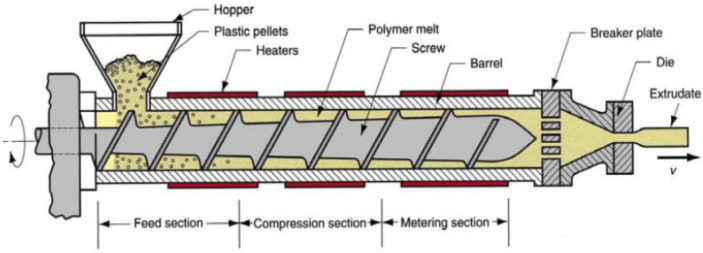
The basic component of an extrusion process [35].

Melt extrusion mixing is the predominant approach to manufacturing thermoplastic polymer-based composites. Two types of extruders can be used, i.e., single-screw extruder (SSE) and twin-screw extruder (TSE), regardless of the number of screws; the main regions of an extruder are the feeding zone (solids conveying), the compression zone (melt), and the metering zone (melt pumping zone) (as reported in Figure 6) [34]:

- *Feeding zone*: in this section, the feeding material, loaded through a hopper, falls by gravity into the heated barrel, contacting the rotating screw. The screw thread, in this zone, is high and constant.
- *Compression zone*: the screw thread reduces and the polymer, advancing along the screw, is forced into a smaller volume. The combination of compression and screw rotation generates friction and, consequently, heat, termed as shear heating. This heating source, along with the heat from the barrel heating system, induces the polymer melting. Moreover, the increase in the internal pressure facilitates the elimination of cavities and gases.
- *Metering zone*: here, the molted polymer is extruded through a nozzle to form the filament. Along this zone, the screw threat is constant throughout its length, analogously to the feeding zone.

SSE is generally used for pure polymers, particularly those characterized by high melt viscosities, as it may produce high pressure inside the barrel during the process [36]. The high pressures and frictions, especially when the screw speed increases, make the SSE process unsuitable for heat-sensitive polymers. The main advantages of this extruder typology are the dimensions and capital investment, which are both remarkably lower compared to TSEs. It is noteworthy that the absence of a shear deformation during the polymer processing in single-screw extruders limits their usage in composite mixing, as it leads to agglomeration and poor dispersion [37]. Despite the poor shear deformations, by carefully tuning the SSE parameters it is possible to achieve a well dispersed material, as demonstrated by the numerous works currently available in the literature. Singh et al. [38] loaded nylon-6 with 40 wt% of (Al + Al_2_O_3_) by using an SSE, and they investigated the effect of powders composition (Al and Al_2_O_3_ ratio), barrel temperature, and die temperature. The reported observations support the conclusion that samples with an Al/Al_2_O_3_ ratio equal to 30/10% and a barrel and die temperature of 175 °C and 195 °C, respectively, exhibit the highest elongation at break and tensile strength. Furthermore, they analyzed the percentage incidence of these parameters over the tensile strength of the final printed material. The authors concluded that the most impactful parameter is the mixture composition (70.3%), whereas barrel temperature and die temperature, respectively, have a 15.2% and 7.3% occurrence. Using SSE, it is common practice to perform multiple extrusions to homogenize the filler in the hosing matrix; nevertheless, as reported by Cieślik et al. [39], who studied the effect of multiple extrusions on PLA/CB composites’ thermal and electrical properties, polymer can also undergo premature degradation at temperatures lower than the tabulated degradation temperature, compromising structural and functional properties; in fact, the authors reported a reduction in the degradation temperature (evaluated by TGA) of 15.7 °C and 11.6 °C after 5× multiple extrusion at 190 °C and 200 °C, respectively. Also, electrical conductivity undergoes a reduction when the system is ×5 reprocessed at 190 and 200 °C, respectively, down to 0.06 S/cm (1.7 times decrease) and 0.03 S/cm (3.7 times decrease).

**Figure 7:**
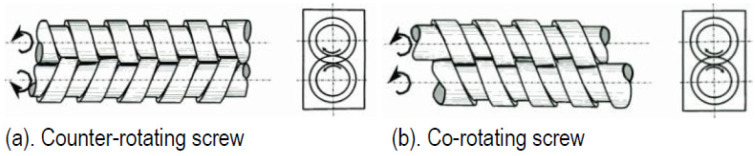
Scheme of extrusion screw type in TSE [40].

In contrast to SSEs, TSEs generate a more intense shear force amongst screws, as well as among the screws and barrel, resulting in superior mixing with uniform filler dispersion. It is noteworthy that due to the intense shear stresses generated by the system, TSEs are not suitable for materials sensitive to shear stresses [40]. Depending on the reciprocal rotation direction of screws, two typologies of extruders can be listed, i.e., counter-rotating and co-rotating screw extruders. Counter-rotating screws are usually used for the profiles extrusion, while the results of co-rotating screws are more suitable for the mixing process [41]. Figure 7 illustrates these two types of extrusion screws.

TSEs are characterized by high mixing and processing capability, are compatible with temperature sensitive polymers [42], and require less power to operate compared to SSEs holding the amount of processed polymer. However, TSEs require large capital investments with higher maintenance costs compared to SSEs [43]. Table 1 summarizes the main advantages and disadvantages of SSEs and TSEs.

**Table 1:** Pros and cons of melt extruder typology.

Extruder Type	Pros	Cons	Reference
SSE	✓ Small capital investment ✓ Low maintenance costs	❖ Poor dispersion ❖ Not suitable for temperature-sensitive polymers	[36,37,44]
TSE	✓ High mixing capability ✓ Better parameter controls ✓ Suitable for temperature sensitive polymers ✓ Lower energy consumption	❖ Large capital investment ❖ High maintenance costs ❖ Not suitable for materials sensible to shear stress	[40,41,42,43]

Although melt extrusion mixing is the primary technique to manufacturing composite filaments for FDM processes, numerous studies use solution mixing to prepare granulates (pellets) for subsequent filament production. This approach is mainly implemented for polymers or reinforcements extremely sensitive to temperature or also when melt mixing is unable to provide a sufficient degree of dispersion [45]. During solution mixing, the filler is dispersed in a polymer solvent by different techniques such as magnetic stirring [46], sonication [47], etc., and the polymer is subsequently dissolved. Once the system is homogeneous, a solvent extraction step is performed and, finally, the dried solid composites are shredded for the subsequent filament making. An example of solution mixing applied to the production of feeding composites for FDM processes is reported by Sakunphokesup et al. [45]. The authors demonstrate that by implementing different mixing techniques (i.e., coagulation method, solution method, and dry method), the highest values of tensile strength and modulus are obtained by solution mixing. This result is likely attributable to the superior dispersion of graphene within the hosting ABS polymer. In another work [48], solvent casting was used to facilitate and enhance the efficiency of PLA/MWCNT composite filament fabrication. In fact, due to the solution mixing pre-stage, the MSCNT dispersion significantly improved, as confirmed by the highest electrical conductivity reaching 2.8 × 10^−7^ S∙cm^−1^ with 5 wt% of filler. Following the mixing of the considered phases, the system is melt extruded to obtain pellets, or shredded to attain granulations. The resulting pellets/granulates undergo an additional process, whose drying time and temperature strongly depend on the specific polymer needed to remove any absorbed moisture before proceeding to the next stage [49].

#### 2.2.2. Filament Shaping and Spooling

**Figure 8:**
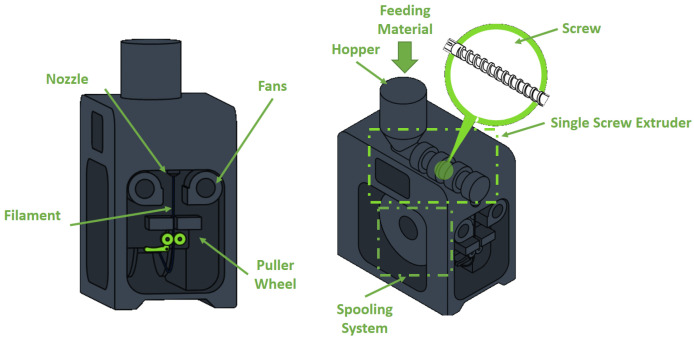
Outline of a filament maker.

In the second step, the feedstock material is shaped to form a filament by an SSE system (Figure 8), loading hopper the base polymer or composite pellets/granulates directly in the. The screw rotation ensures system melting and promotes the homogenization of the composite material. As the temperature inside the barrel influences the rheological behavior of the molten polymer, it becomes clear that the uniform diameter of the filament is determined by an optimized temperature profile inside the extruder. Also, the screw speed strongly affects the filament quality and diameter, as an increase in the flow rate causes too rapid a solidification, leading to internal porosities.

The semi-liquid filament exiting the extruder is drawn by puller wheels to achieve a precise diameter. The pulling speed is a key factor in determining the filament’s thickness, with slower speeds resulting in larger diameters and vice versa. Commonly, filament-making equipment incorporates thickness optical sensors whose real-time signal is retrofitted to the motherboard. According to the sensor measurement, the pulling speed is adjusted to attain the set filament diameter (generally 1.75 or 2.85 mm).

External fans are generally used to cool the extracting filament, with higher fan speeds leading to an increase in diameter. The final stage of the filament-making process includes winding the wire around a spool, ready for printing [5].

## 3. Properties and Applications of FDM Polymer Composites

**Figure 9:**
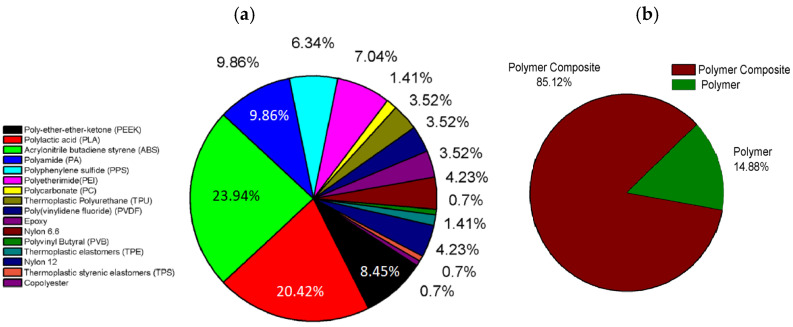
Percentage utilization of (**a**) common polymers and (**b**) polymers/polymer composites used for FDM in published work from 2000 to 2020 [51].

Currently, two main typologies of materials are used in the majority of FDM processes, i.e., thermoplastic polymers and thermoplastic composites. Among the thermoplastic polymers, used in the forms of pure polymers or matrix, the most commonly used are ABS, PLA, polycarbonate (PC), PEEK, polyether imide (PEI), PVA, and polyamide (PA—e.g., Nylon6, Nylon12). Figure 9a lists some of the used polymers in FDM processes as a function of their usage percentage. Clearly, ABS and PLA contribute as major polymers [50].

Although pure thermoplastic polymers are used in different application fields, the use of composites (see Figure 9b) is mainly driven by the final performance of the FDM manufacts. As matter of fact, pure thermoplastic printed parts exhibit ineffective mechanical properties (compared to traditional manufacturing techniques) due to degradative phenomena during printing, which could result in chain scission, weaker molecular bonding, and molecular structural variations in the final processed material [52,53]. To address these limitations, the primary strategy consists of blending the polymer matrix with a reinforcement, enhancing not only the mechanical and thermal properties but also inducing novel features, such as electrical conductivity, electro magnetic interference (EMI) shielding, and antibacterial properties [54].

### 3.1. ABS-Based Composites

ABS is a terpolymer made of styrene, acrylonitrile, and polybutadiene. It is generally used in household items and toy manufacturing (LEGO^®^ bricks are made of ABS [55]) due to its relatively low harmful effects on human health compared to other polymers [56]. ABS is an amorphous, glassy, tough, and impact-resistant polymer, characterized by sufficient melt fluidity and, consequently, printability [12], and these features have made it one of the most popular choices of polymer for FDM process. Despite this, the mechanical properties of ABS FDM-printed parts are remarkably lower compared to those manufactured by traditional manufacturing processes. In addition, the layer-by-layer nature of FDM processes induces an intrinsic anisotropy in the printed part properties; in this regard, Ahn et al. [57] compared the tensile strength of injection molded (IM) ABS- and FDM-printed ABS with different raster angles. The reported results reveal that the measured tensile strength values for crisscross raster [±45°] and cross raster [0/90°] printed samples are between 65% and 72% with respect to IM ABS. Moreover, as also reported, air gaps among the raster fibers have a relevant effect on tensile strength. Negative gap values (−0.075 mm) increase the tensile strength (16 MPa) compared to zero air gap (11.5 MPa) with a crisscross raster.

To improve mechanical properties and mitigate anisotropy, ABS is loaded with different reinforcements. Le et al. [58] have shown that multiwalled CNT (MWCNT)/ABS filaments with excellent filler dispersion can be manufactured by single-screw extruders. The authors used six different filler concentrations, and for the highest, i.e., 4.0 wt%, they were also able to print dogbone samples for tensile testing. The best result in terms of tensile strength was measured for the 2.0 wt% filled samples, with a 41.8% increase compared to the unmodified system.

**Figure 10:**
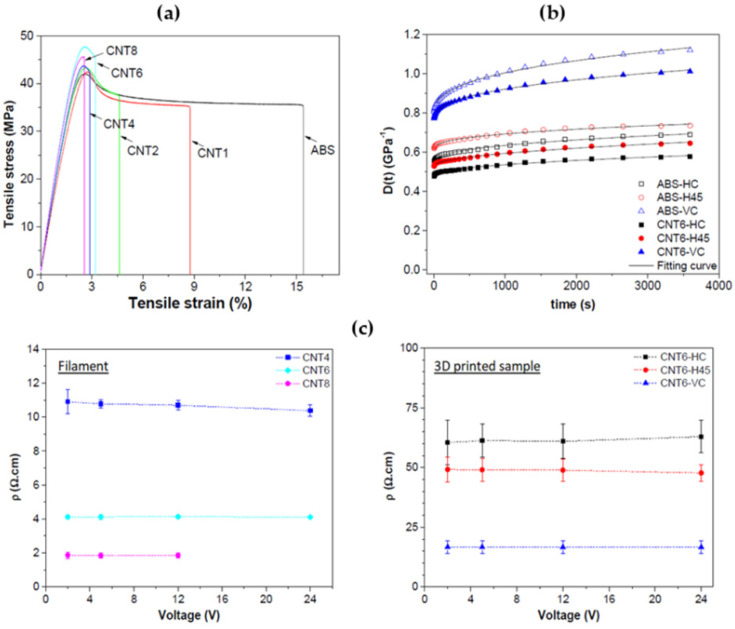
(**a**) Tensile stress vs. strain curves for ABS/CNT filament; (**b**) creep compliance for ABS/CNT printed samples; (**c**) electrical resistivity of ABS composites [59].

Dul et al. [59] developed CNT-loaded ABS composites, implementing a solvent-free approach to study the mechanical and electrical properties as a function of CNT content and printing orientation; using melt extrusions by TSE, the authors ensured a homogeneous dispersion of CNT in the filament, and consequently in the printed parts. The optimization, in terms of filler concentration, was performed based on the tensile property values of the extruded filament, as reported in Figure 10a, and the optimal balance between tensile modulus and strength results with 6 wt% of CNT (CNT6). CNT6 filament was printed using different printing configurations (flat-concentric, flat-45° and vertical, upright concentric), and the creep results have shown that CNTs have a positive effect on long-lasting loads due to the creep compliance reduction (both in terms of elastic and visco-elastic contribution—see Figure 10b), regardless of the printing orientation. In addition, electrical characterization of the printed parts has shown an actual reduction in resistivity with CNT (for contents higher than 2 wt%). It is noteworthy that the increase in conductivity is more pronounced in the filament composite compared to composite printed samples, and this is attributed to a poor raster fiber interphase, acting as a resistance among the fibers. The incorporation of fillers into in ABS can be a laborious process, which requires the addition of specific plasticizers to facilitate the correct filament extrusion and subsequent winding. This challenge was encountered by Zhong et al. [60], who developed FDM composites based on ABS reinforced with short glass fibers (SGFs) to enhance matrix hardness and strength. The short fiber addition induced a remarkable improvement in ABS strength, although reducing flexibility and processability. To overcome this later effect, authors added LLDPE as a plasticizer and hydrogenated Buna-N as a compatilizer (between ABS and LLDPE), achieving more interesting results in the case of 18%wt SGF, with a remarkable increase in tensile strength (i.e., 58.60 MPa) compared to the unmodified ABS/LLDPE blend (i.e., 24.50 MPa).

The use of natural fillers has always been an attractive field of research to develop innovative micro/nanocomposites. Ahmad et al. [61] produced ABS/oil palm fibers (OFMs) to improve FDM ABS mechanical properties by using natural materials derived from waste industrial processes. Different contents of OFM were considered (3, 5, and 7 wt%), inducing a slight increase in tensile properties (strength: +6.9% and modulus: +4.5%) and impact energy (+3.2%). Nevertheless, a further increase in OFM content weakens the fiber/matrix interface, mainly due to voids and cluster formation, resulting in an abrupt reduction in the abovementioned material performance. This work also revealed a reduction in moisture absorption due to the fibers hindering the water molecules’ permeation. Also, Torrado Perez et al. [62] investigated the effect of a natural jute fiber at 5 wt% on the mechanical properties of FDM ABS polymer. The results confirmed a reduction in the tensile strength (−9%), with an increase in both elastic modulus (+0.9%) and flexural strength (+42.9%). In addition, the jute fibers composites showed a ductile fracture attributable to a transfilament rupture mechanism caused by voids formed during the 3D printing process due to the fiber degradation with gas formations. This phenomenon starts at 180 °C [63], which is a lower temperature compared to the processing temperature of ABS.

**Figure 11:**
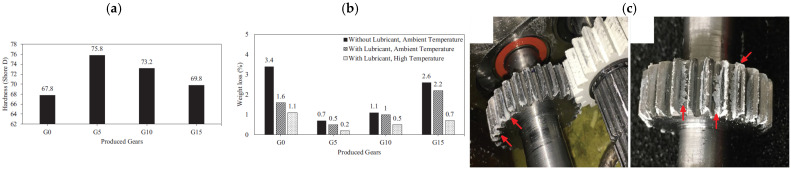
(**a**) Hardness as a function of the glass fiber content; (**b**) weight loss after gear work as a function of the glass fibers content in different working environments; coupled steel and FDM-(**c**) printed gears and effect of abrasion on the steel gear (red arrows indicate the polymers scraps on the steel gear after gears work) [65].

As previously mentioned, FDM manufacts are characterized by inferior mechanical properties compared to those produced by traditional processes; however, their manufacturing and maintenance costs are remarkably lower. The addition of suitable fillers potentially enables the production of FDM manufacts with mechanical properties comparable, or superior, to those of IM parts. Meng et al. [64] reported how the addition of nano-montmorillonite narrows the disparity between the mechanical properties of FDM and IM specimens, with an increase in tensile strength (42 MPa for IM specimen) and flexural strength (72 MPa for IM specimen) from 29.5 MPa to 37.1 MPa and from 54.8 MPa to 64.2 MPa, respectively. Moreover, printing the specimens with two different orientations, the authors reported a reduction in anisotropy with the addition of CaCO_3_. Specifically, the percentage variation in tensile strength between flat-printed and upright-printed specimens resulted 42.1% for pure ABS and almost halved (i.e., 23.9%) for ABS/CaCO_3_ specimens. Another example of FDM composites that exhibit superior mechanical properties compared to their IM counterparts was reported by Bodaghi et al. [65], who developed milled glass fiber-loaded ABS filament to print gears through the FDM process. Glass fibers were assessed to improve the wear properties of the gears, whereas for high filler content the wear resistance tends to decrease due to voids and cluster formation. The improvement in wear properties, reported in both dry and lubricated environments, is mainly attributable to the hardness increase in the material due to the fibers’ presence, as reported in Figure 11a. The authors also demonstrated an improvement in the wear resistance at higher temperatures, such as at 100 °C, compared to the unmodified matrix, likely related to the increase in the glass transition temperature (Tg) due to the glass fibers’ presence. As reported in Figure 11b, the wear resistance of the gear was measured, evaluating the weight loss during the gear working, and in Figure 11c the effects of the abrasion between the steel and FDM-printed gear tooth are indicated.

**Figure 12:**
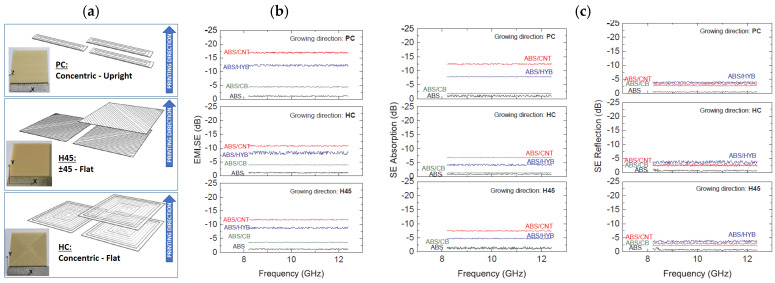
(**a**) Printing configurations of ABS/CNT, ABS/CB, and ABS/HYB; (**b**) absolute EMI SE and (**c**) EMI SEA/EMI SER for ABS/CNT, ABS/CB, and ABS/HYB [66].

Polymer reinforcement not only affects the mechanical properties of the hosting matrix but can improve also other functionalities, as reported by Schmitz et al. [66]. In this work, ABS was filled (3 wt%) with CNT, carbon black (CB), or a combination thereof (HYB) to study the induced changes in terms of mechanical, electrical, and EMI shielding properties. Tensile tests performed on ABS/CNT filaments revealed an increase of approximately 10% for both tensile strength and modulus. Regarding filaments’ electrical conductivity, the value increased from 10^−15^ Scm^−1^ to 10^−2^ Scm^−1^, respectively, in the case of pure ABS and ABS/CNT. The authors also analyzed the EMI shielding properties of the printed nanocomposites, observing that this property is significantly affected by the printing configuration (see Figure 12a). As shown in Figure 12a, the highest EMI shielding effectiveness (SE), i.e., −16 dB, was observed for samples loaded with CNT and printed using the concentric upright configuration (labeled PC). Since the mechanisms responsible for the attenuation of the incident wave through a specimen are reflection (SER) and absorption (SEA), the authors accounted their relative contribution to total EMI SE, finding that the absorption is the predominant mechanism of shielding. Table 2 summarizes literature results related to the ABS based nanocomposites for 3D printing.

### 3.2. PLA-Based Composites

PLA is a thermoplastic aliphatic polyester derived from lactic acid polymerization. Lactic acid synthesis involves the microbial fermentation of sugars, which in turn are produced by sustainable and renewable feedstock such as starch, plant crops, and sugarcane. The main advantages of PLA compared to other non-biodegradable and fossil-derived polymers, such as polyethylene and polypropylene, include its biodegradability, bio-absorbability, and renewable source origin [70]. PLA is characterized by mechanical properties relatively superior to other traditional polymers, and it therefore finds widespread use in different polymer application fields, including textiles, chemical industries, and automotive composites as well as in feedstock material in FDM processes [71]. Furthermore, PLA transparency is high (comparable with PS and PET) and its cytotoxicity is low, and for these reasons this polymer finds suitable applications in biomedical and food packaging [72] sectors. Nevertheless, the main drawbacks of PLA are high brittleness, low electrical conductivity, and poor thermal properties, which limits the possibility of expanding PLA’s usage to more advanced and valuable applications, such as aeronautics and electronics. In this regard, it is a common strategy to improve PLA properties by adding different plasticizers and fillers [73]. An initial attempt to enhance PLA’s mechanical properties was reported by Zhang et al. [74], considering metal fibers as filler. Aluminum fibers (7 wt%) were dispersed in a PLA matrix and later tested in terms of dynamical mechanical and tensile properties. The authors reported an increase in both storage modulus (+23.6%) and Tg (+0.86 °C), indicating that the thermal resistance capacity of PLA was enhanced by the Al fibers’ presence, whereas both the tensile strength (−16.6%) and modulus (−1.71%) were resultingly lower compared to bare PLA; however, at the same time, elongation to break (+6.7%) was higher, and the higher elasticity and lower tensile strength of Al fibers can explain this phenomenon.

Among the different fields of FDM processes, the biomedical area is considered the most interesting, in particular in the development of scaffolds for bone tissue engineering substitutes. Since hydroxyapatite (HA) is a fundamental bioactive material for bone graft substitutes, the high loading of HA composite scaffolds is extremely important to achieve osteoconductive and good osteointegration properties. Corcione et al. [75] attained this incorporation by using a twin-screw extruder and up to 50 wt% of HA in a PLA matrix, which is a value remarkably higher compared to other works (max 30 wt%) [76]. Two main features associated with the presence of HA were recorded: (1) up to 30 wt%, HA acts as nucleating agent, as indicated by XRD analysis; (2) although in the FDM process the infill density was set to 100%, the porosity of the sample with higher HA content (i.e., 15–50 wt%) resulted in approximately 18%, indicating that powder induces porosity during the FDM extrusion process, independently of the filler content.

**Figure 13:**
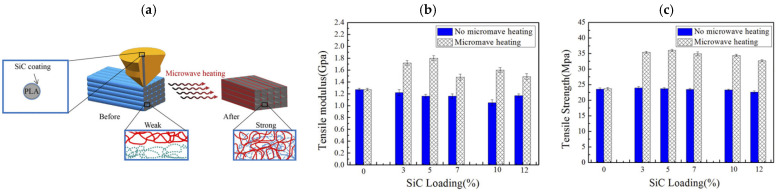
(**a**) Interface fusion after microwave post-treatment in SiC/PLA 3D parts obtained by FDM (blue and red lines represent the polymeric chains belonging to two adjacent raster fibers); (**b**) tensile modulus and (**c**) strength of the untreated and microwave treated samples [77].

In FDM processes, many strategies are implemented with the aim of improving the mechanical properties of the raster fibers’ interphase. SiC is a microwave absorber that converts the microwave (frequency range 8.2–12.4 GHz) into heat in a short time, and for this reason Wang et al. [77] developed SiC-coated PLA filaments as innovative FDM feeding wires. Upon printing, the FDM manufact undergoes a microwave post-process to promote the melting of the raster fiber interphase (Figure 13a), remarkably increasing tensile modulus (Figure 13b) and strength (Figure 13c). The presence of SiC not only induces melting in PLA but also promotes recrystallization, acting as a nucleating agent. The author pointed out that an excess of SiC (up to 12 wt%) could detrimentally affect the interlayer bonding following the microwave post-treatment due to separation effects.

Synergic effects on thermal and electrical conductivity in FDM polymer composites are observable using carbon-based fillers with a different dimensionality (2D or 1D) and aspect ratio. GNPs (2D) and MWCNTs (1D) in bi-filler systems tend to form denser percolative networks within the hosting matrix, leading to enhanced thermal and electrical conductivities compared to mono-filler systems holding the same filler content [71].

**Figure 14:**
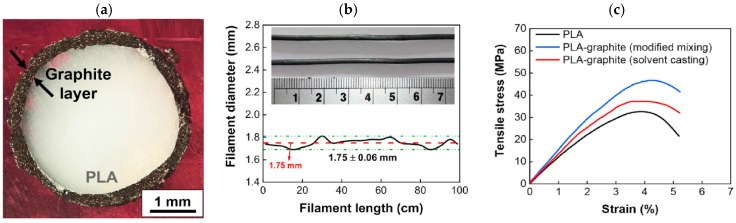
(**a**) Cross-section of PLA/graphite pellet; (**b**) filament diameter variation in the composite filament produced via modified solvent casting method; (**c**) tensile stress–strain curves of 3D-printed PLA-graphite composites [78].

As previously stated, for FDM processes, implementing mixing to attain feedstock filaments is fundamental. Zerankeshi et al. [78] realized graphite-based feeding filaments by using two similar mixing techniques: (1) a solvent casting method, where PLA and graphite are directly mixed in the presence of a large amount of dichloromethane (DCM), and (2) a modified solvent casting method, where graphite is first dispersed in a small amount of DCM and then added to PLA pellets, leading to the formation of a core/shell pellet where a graphite/PLA shell surrounds a pure PLA core, as reported in Figure 14a. The use of a small amount of solvent ensures its complete evaporation, leading to a homogeneous filament, both in terms of graphite dispersion and filament thickness (Figure 14b), which strongly affects the FDM process, with a significant improvement in system mechanical properties, as reported in Figure 14c.

To attain suitable EMI shielding features in FDM materials, GNPs are generally the most relevant candidates. It was reported [79] that samples printed using 10 wt% GNP-loaded PLA filament show a value of EMI SE equal to −16 dB in the X-band frequency range (8–12 GHz), which makes them suitable for the production of lightweight EMI shielding devices.

**Figure 15:**
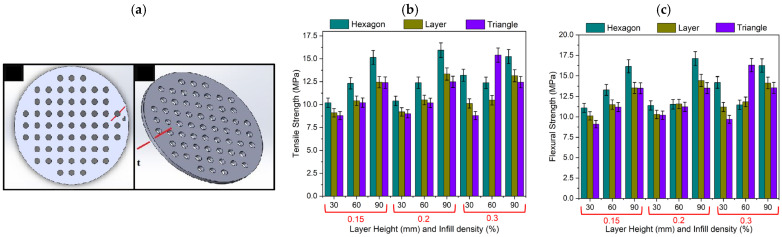
(**a**) MPP printed by FDM process; (**b**) tensile and (**c**) flexural strength of samples printed with different configuration, infill density, and layer thickness [82].

Due to the sustainability of PLA, considerable research efforts have been devoted to realizing PLA-based nanocomposites that are fully biodegradable using green fillers. Coppola et al. [70] developed PLA composites by loading the matrix with hemp fibers (up to 5 wt%), achieving a 65% increase in tensile modulus compared to unmodified PLA. Natural fibers are characterized by superior acoustic properties due to their lower densities and higher porosities (compared to synthetic fibers), allowing for better sound absorption and dissipation [80]. Moreover, FDM is known to be particularly efficient in the production of complex geometries, like those needed in micro-perforated panels’ (MPPs—see Figure 15a) production, i.e., devices used in sound absorption. Vigneshwaran et al. [81] realized MPPs by employing FDM processes fed with PLA/wood fibers filaments. The results show that the sound absorption coefficient increases linearly with hole diameter (d); moreover, specimens printed with a hexagonal configuration, 0.2 mm layer thickness. and 90% infill density are characterized by the largest tensile (Figure 15b) and flexural strength (Figure 15c) values. Table 3 summarizes literature results related to the PLA based nanocomposites for 3D printing.

### 3.3. PEEK-Based Composites

Polyetheretherketone (PEEK) is a high performance semi-crystalline thermoplastic polymer which finds applications in many fields, such as biomedical (bio implants [84]), aerospace (satellites and thermal shields [85]), chemical (micro and macro-reactors [86]), and electronic [87]. Despite its cost, the use of PEEK is growing due to many interesting features, such as the following [88]:

- low density (1.32 g/cm^3^),
- good mechanical properties (tensile failure strain: 15%, good impact strength),
- preservation of mechanical properties up to 250 °C,
- excellent resistance to chemicals, solvents and hot water,
- biocompatibility.

PEEK is characterized by a structure containing an equal balance of a ketone group, which provides rigidity, and an ether group, related to flexibility. Both linkages enhance the melt processability, allowing PEEK to be considered in many manufacturing processes, such as injection molding, compression molding, and FDM [89]. The addition of nanofiller further enhances the superior properties of PEEK; it is noteworthy that the use of natural fibers is not compatible with the very high processing temperature of PEEK, which is generally higher than 320 °C.

As for other polymers, the mechanical properties attained for 3D-printed PEEK elements are generally lower compared to traditionally processed PEEK manufacts, primarily due to porosity level and low interfacial strength. Nevertheless, Golbang et al. [90] demonstrated that the addition of WS_2_ fullerene particles to PEEK (1 wt%) enables the 3D printing production of PEEK manufacts characterized by mechanical properties similar to those of unmodified PEEK produced by IM. The authors relate this behavior to the reinforcing and lubricating effect of WS_2_, which allows for higher molecular inter-diffusion and better layer bonding. Since HA is the main bioactive material for bone grafts substitutes and considering PEEK’s biocompatibility and mechanical properties similar to those of the human femoral cortical bone, Rodzen et al. [91] prepared PEEK/HA nanocomposites (up to 30 wt% of HA) suitable to develop bone prosthesis by FDM processes. Filament production was performed by using a twin-screw extrusion, which ensured that HA was evenly distributed throughout the bulk and across the surface of the 3D-printed samples. The uniform distribution of the filler within the matrix bulk induced a remarkable increase in the PEEK mechanical properties (elastic modulus: +44%, flexural modulus: +33%), while the uniform distribution on the prosthesis surface provided enhanced osseointegration to ensure more rapid, improved, and stable fixation with bone tissue.

**Figure 16:**
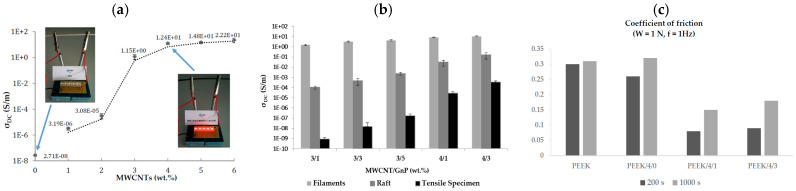
(**a**) Electrical conductivity of PEEL/MWCNT filaments as a function of CNT content; (**b**) comparison between electrical conductivity of filament, raft, and 3D-printed samples as function of filler concentration; (**c**) coefficient of friction of PEEK and PEEK/MWCNT/GnP filaments [92].

Electrically conductive PEEK-based filaments can be attained by tailoring carbon fillers, as reported by Gonçalves et al. [92]. Binary and ternary nanocomposites based on GNP- and/or MWCNT-filled PEEK have reported interesting results. Electrical conductivity tests on “ad hoc” manufactured filaments have revealed a percolation threshold between 2 and 3 wt% of CNTs (Figure 16a), and the combination with GNPs can further enhance the electrical conductivity up to 10 S/m. FDM-printed dogbone samples showed an increased modulus and ultimate tensile strength, but the elongation at break was lower compared to the extruded samples due to the intrinsic porosity of the FDM manufacts. The porosity level of 3D-printed parts also affects the surface and volumetric contributions to the transport properties, and this reasonably justifies the electrical conductivity loss of printed systems compared to the extruded element (Figure 16b). Graphite’s lubricating effect is highlighted by the tribological tests performed on the filaments, with the coefficient of friction almost halved in presence of 1 wt% of GNPs, compared to neat polymer; on the other hand, CNTs do not show any effect on the friction coefficient (Figure 16c).

**Figure 17:**
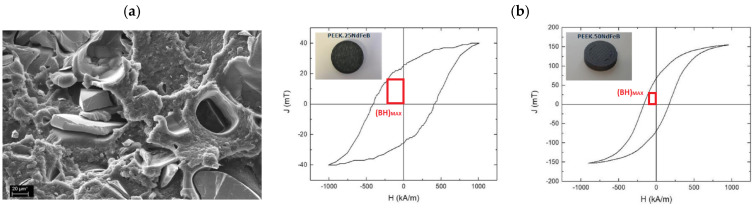
(**a**) Fracture surfaces of PEEK_50NdFeB; (**b**) hysteresis loop for PEEK_25NdFeB (**left**) and PEEK_50NdFeB (**right**) printed magnet [94].

As already reported, PEEK is an excellent candidate for high-performance applications; one example was proposed by Pigliaru et al. [93], reporting the 3D printing of PEEK-NdFeB nanocomposites to develop Permanent Rare Earth composite magnets to use in efficient motors and generators featuring a high energy density. Three different NdFeB concentrations were employed, i.e., 25 wt%, 50 wt%, and 75 wt%, in a combined mixing/filament production performed by a single-screw extruder. Despite the uniform filler dispersion reported by the authors, the tensile tests show a remarkable drop in tensile strength (−23%) associated with the poor filler/matrix interface and irregular NdFeB edges acting as initiation sites for damages and cracks (Figure 17a). The magnetic measurements (Figure 17b) show a maximum energy product, (BH)_MAX_, of 130 J/m^3^ for PEEK_25NdFeB and 750 J/m^3^ for PEEK_50NdFeB, demonstrating the feasibility of the magnetic 3D-printed composite with PEEK. It is noteworthy that the experimental (BH)_MAX_ values were lower than theoretical predictions, likely due to the intrinsic porosity of the FDM magnets. A further innovative application of the PEEK system was proposed by Wu et al. [94], employing PEEK/boron carbide (BC) nanocomposites for nuclear components. BC is a well-known neutron absorber that, combined in different concentrations (i.e., 10, 20, and 30 wt%) with PEEK, allows for the 3D production of efficient and customizable neutron shields capable of preserving mechanical properties at high temperatures (measured heat deflection temperature >300 °C). It was demonstrated that the addition of BC not only improved the mechanical properties of the hosting matrix (tensile strength, +27.3%; flexural strength, +66.3%) but also induced remarkable neutron shielding effectiveness. An 80 mm thick PEEK/BC shield, filled at 20 wt%, can absorb up to 80% of the neutrons generated by the radiation source. Table 4 summarizes literature results related to the PEEK based nanocomposites for 3D printing.

**Table 4:** FDM PEEK-based composite properties.

Matrix	Filler (Content)	Δσ^T^ [%] (PD–PO)	ΔE [%] (PD–PO)	Δε [%] (PD–PO)	Δσ^f flex^ [%] (PD–PO)	ΔE^flex^ [%] (PD–PO)	ρ_3D_ [Ωcm] (PD–PO)	REF
PEEK	WS_2_ fullerene (1 wt%)	+33 (±45–F)	+11 (±45–F)	-	-	-	-	[90]
PEEK	HA (30 wt%)	+2.2 (±45–F)	+44 (±45–F)	−30 (±45–F)	−16% (±45–F)	+33 (±45–F)	-	[91]
PEEK	MWCNT/GNP (4/3 wt%)	+1.2 (±45–F)	+15 (±45–F)	−6.9 (±45–F)	-	-	10^−1^ (±45–F)	[92]
PEEK	NdFeB (25 wt%)	−26 (±45–F)	−14 (±45–F)	−43 (±45–F)	-	-	-	[93]
PEEK	BC (10 wt%)	+27 (0/90–F)	+66 (0/90–F)	-	-	-	-	[94]
PEEK	GNP (3 wt%)	−0.15 (0–F)	+16.8 (0–F)	11.6 (0–F)	-	-	-	[95]

### 3.4. PVA-Based Composites

Poly(vinyl alcohol) (PVA) is a synthetic multi-hydroxyl polymer widely employed in biomedical tissue engineering due to its low cost and excellent properties, such as solvent resistance, biocompatibility, hydrophilicity, and semi-permeability, which all are features required for cell growth and survival [96]. PVA is also known to be one of the few water-soluble polymers susceptible to biodegradation in the presence of acclimated microorganisms. Nevertheless, due to its hydrophilicity, PVA shows a strong tendency to water sorption, with consequent detrimental effects on mechanical performances, which inherently limits its use for some desired applications [97].

**Figure 18:**
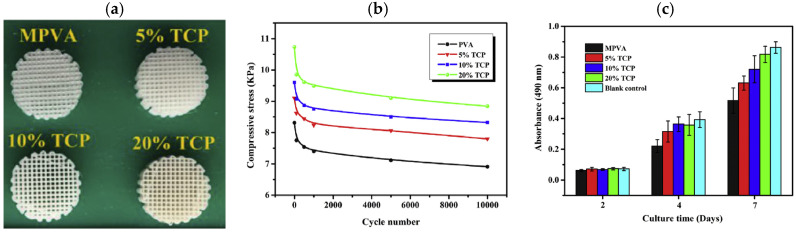
(**a**) Optical micrographs of the FDM scaffolds; (**b**) maximum stress at 40% strain, evaluated in cyclic compression tests performed on the composite scaffolds; (**c**) MTT assays of cells cultured on the scaffolds after 2, 4, and 7 culture days [99].

In recent years, due to the growing interest in additive manufacturing technologies, PVA has been reconsidered due to some specific features, such as flowability and extrudability, critical for 3D printing feedstock materials. The use of PVA in 3D printing processes finds remarkable interest in tissue engineering due to a combination of biocompatibility, control accuracy in scaffold structure, dimensions, and porosity, which enables the production of architectures that mimic the functionalities of biological tissues [98]. Within this framework, polymer/bioceramic composite scaffolds are considered as promising biomimetic substitutes for bone tissue engineering due to their tailored mechanical properties and improved bioactivity. Chen et al. [99] developed PVA/β-tricalcium phosphate (β-TCP) composite filament, with different β-TCP contents (0–20 wt%), to realize polymeric scaffolds with enhanced mechanical and bioactivity features by FDM (Figure 18a). The results show that the homogeneous bioceramic dispersion, attainable by a solid-state shear milling, potentially lead to mechanical performance improvement, especially in terms of compression fatigue behavior (Figure 18b). The cytotoxicity of the scaffolds was assessed by using the MTT assay upon days 2, 4, and 7 of cell culture and, as is clearly reported in Figure 18c, it was found that only upon days 4 and 7 of cell culture did the 20 wt% β-TCP loaded scaffold show significantly higher cell proliferation compared to the day 2 culture; this evidence suggests that β-TCP filler acts as an excellent cell growth factor. Another bioceramic commonly employed in tissue engineering is represented HA; in particular, PVA/HA shows excellent mechanical properties and osteoconductivity, enabling cartilage repair. Nevertheless, the HA content induces the formation of PVA foam during the extrusion, hindering the formation of a uniform thickness filament for FDM processes. Wu et al. [100] proposed a novel strategy to prevent this issue, implementing a modification of HA by 3-glycidoxypropyltrimethoxysilane (KH560), and later mixing the attained modified particles with PLA before blending and extruding the HA/PLA nanocomposites to achieve a ternary system. It was found that the modified HA segregates into PLA, preventing foam generation. This approach enables the manufacturing of smooth and homogeneous 1.75 mm thick filaments and thus the correct printing of a suitable scaffold architecture for tissue engineering.

**Figure 19:**
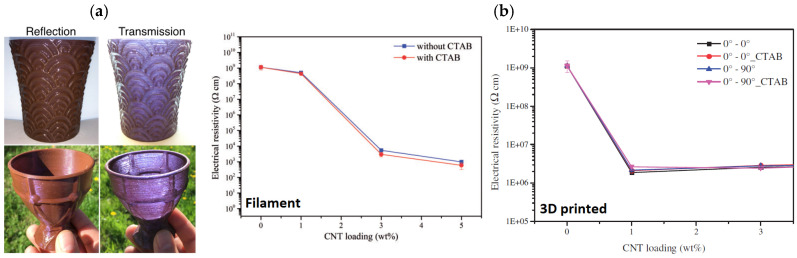
(**a**) PVA/gold NP 3D-printed cups showing the dichroic effect [104]; (**b**) comparison between electrical resistivity of PVA/CNT filament (**left**) and 3D-printed samples (**right**) as a function of CNT content [102].

One of the primary advantages of using PVA in the development of 3D printed nanocomposites is related to its water solubility. Indeed, fillers, which are only synthesizable or soluble in water present serious issues if dispersed in traditional polymers (e.g., PLA and ABS) without employing co-solvents; conversely, when using PVA as a matrix, no additional solvent is necessary. Kool et al. [101] demonstrated this by incorporating gold nanoparticles (Au NPs) into PVA using water as solvent, fabricating FDM artifacts exhibiting a dichroic effect (Figure 19a). The dichroic effect is inherently associated with the dimensions (larger than 50 nm) and shape (elliptical, with a mean aspect ratio of 1.4) of the synthesized Au NPs. Chloroauric acid served as the Au NPs precursor and, upon synthesis, PVA powder was directly added to the synthesis solution to attain PVA/Au NP nanocomposites. Ultimately, the dried system was shredded and then extruded to form ready-to-print filament. Another example of PVA-based nanocomposite produced by solution mixing was reported by Rigotti et al. [102]. The author incorporated CNT in PVA, directly mixing the carbon nanofillers within the polymer solution and, upon water evaporation, extruding it by a single-screw extruder. The results indicate that the use of a surfactant, like hexadecyl trimethyl ammonium bromide (CTAB), detrimentally affects the mechanical and thermo-mechanical CNT-related improvement. Moreover, although both filament and 3D-printed samples show a reduction in electrical resistivity compared to pure polymer, the FDM process limits the formation of an efficient percolative path for electron conduction, and consequently the electrical resistivity of 3D-printed sample is higher than that of the extruded filament with same CNT content (Figure 19b).

As previously reported, carbon nanomaterials are commonly employed to induce EMI shielding properties in the hosting matrix. Yang et al. [103] developed PVA/GNPs filaments to print nanocomposites with enhanced EMI shielding properties by solution mixing. PVA is characterized by melting and decomposition temperatures that are very similar due to its high crystallinity and strong hydrogen bonds, which poses critical difficulties for the thermal process to occur, and for this reason glycerol was added to the system to improve polymer processability, reducing the hydrogen bonding strength and crystallinity level. The addition of 8 wt% of GNPs improves the mechanical properties of PVA, yielding to a Young’s modulus of 49.1 MPa, an ultimate tensile stress of 10.6 MPa, and an elongation at break of 128.4%. Furthermore, 2.43 mm thick samples record 26–32 db EMI SE, and this is a suitable level for practical application in telecommunication.

### 3.5. Thermoplastic Polyurethane (TPU)

**Figure 20:**
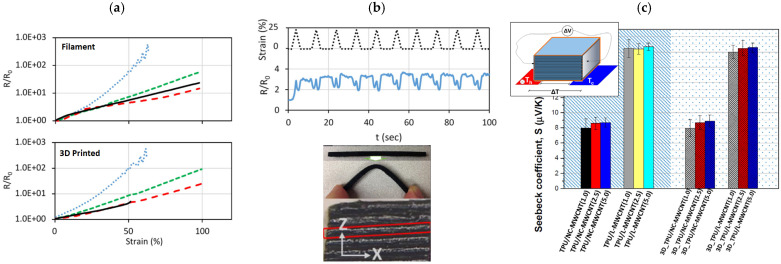
(**a**) Normalized resistance plots of extruded filaments and 3D-printed TPU/MWCNT nanocomposites as a function of strain during loading; (**b**) cyclic strain plots of FDM 3D-printed TPU/2 wt% MWCNT [108]; (**c**) Seebeck coefficient of TPU/NC-MWCNT and TPU/L-MWCNT nanocomposites [107].

In addition to the aforementioned polymers, others thermoplastic polymers are used as FDM feeding material. Thermoplastic polyurethane (TPU) is one of the most affordable soft polymers available for FDM techniques. Compared to other soft polymers, such as PDMS and hydrogels, TPU reports superior strength and toughness, as well as enhanced resistance against oil, cold, and aging, along with good abrasion resistance and high elongation to break [104]. Due to TPU’s soft nature, multiple attempts have been made to use this polymer to fabricate 3D-printed piezoelectric [105,106] and thermoelectric [107] devices, also compensating its poor electrical conductivity with carbon-based filler. Christ et al. [108] considered MWCNT to 3D print highly elastic strain sensors, demonstrating that nanofiller addition increases material electrical conductivity, strength, and elastic modulus, with this later significantly enhancing the printing capability by reducing the material instability under self-weight load. Normalized resistance (ΔR/R_0_) plots as function of the applied strain (Figure 20a) show similar values for filament and 3D-printed samples, indicating that the FDM process does not degrade the piezoelectrical behavior of the material (excellent raster fiber interpenetration during printing). At a lower MWCNT content (2 wt%), a remarkably higher ΔR/R_0_ value (at 50% fixed strain) is recorded compared to higher concentrations, indicating greater sensitivities. Therefore, the MWCNT content enables the tuning of the printed piezoelectric device’s sensitivity for specific applications. Cyclic strain tests show a very good device response (Figure 20b).

TPU was also employed to print thermoelectric devices by Tzounis et al. [107], investigating the effect of MWCNT with different morphologies (NC- and L-MWCNT) and concentration levels (1 wt%, 2.5 wt%, and 5 wt%). It was found that a 2.5 wt% concentration represents an optimized level of MWCNT in terms of electrical conductivity (σ > 100 S/m), still showing a higher improvement in terms of mechanical property enhancement (ΔE = +180%, Δσ^T^ = −27%, ε = −7%). The authors also indicated some interesting results related to the Seebeck coefficient (i.e., the ratio between the measured thermoelectric voltage and the applied temperature difference), as follows:

- while MWCNT morphology slightly affects the mechanical and electrical properties of printed samples, the Seebeck coefficient is remarkably affected by the CNT typology (Figure 20c);
- the printing process does not influence the Seebeck coefficient, as the values are similar for filaments and 3D-printed samples (Figure 20c);
- the 3D-printed samples are isotropic in terms of the Seebeck coefficient.

These features make TPU/MWCNT an excellent feedstock material for the fabrication of flexible and stretchable organic thermoelectric generators using the 3D FDM printing technique.

The same system, TPU, was suggested to fabricate wearable strain sensors by the FDM process. Li et al. [109] filled TPU with MXene (2D filler), MnFe_2_O_4_ (0D filler), and MWCNTs (1D filler) to print wearable sensors and then opportunely calibrated them to detect fingers, knees, and wrist flexion, as well as throat movements during speaking and swallowing. These devices, characterized by a Gauge Factor (GF) ranging from 1.33 to 3.73, report a durable behavior (more than 6000 cycles with different speeds) and show EMI shielding properties with an effectiveness approximately of 31.2 dB with a 2.1 mm printed thickness.

Despite their beneficial features, TPU systems show several drawbacks, including poor shape stability and low mechanical strength. The available literature reports a different strategy to overcome these limitations. Larazza et al. [110] developed waterborne polyurethane-urea (WBPUU) filaments loaded with graphene at 3wt%, which improved the printability due to induced stiffening effect, with consequent enhancement of the mechanical properties (ΔE = +429%, Δσ^T^ = +377%, ε = +21%) and thermal stability. Moreover, printed WBPUU/graphene nanocomposites show biocompatibility, making them an excellent candidate for flexible filaments in biomedical applications. Mrówka et al. [111] analyzed the cytotoxicity of developed TPU base nanocomposites filled with Halloysite nanotubes (HNTs), which do not have toxic effects on normal cells according to MTT cytotoxicity tests. In addition, HNT at 2 wt% in a TPU matrix leads to a significant improvement in the tensile properties (ΔE = +16%, Δε = +50%), hardness (+1.3%), and coefficient of friction (−12%) compared to neat TPU.

### 3.6. Polyetherimide (PEI)

**Figure 21:**
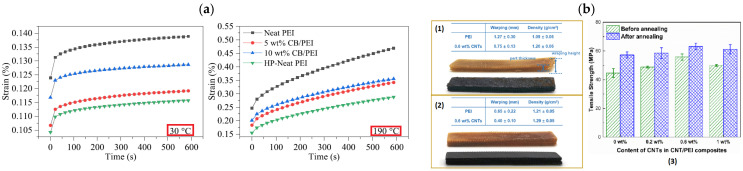
(**a**) Creep strain curves of PEI/CB samples at 30 °C and 190 °C [112]; effect of annealing treatment on ±45 PEI/CNT (0.6 wt%) printed samples on warping and tensile strength: warping (**b.1**) before and (**b.2**) after annealing; (**b.3**) tensile strength results [115].

Amorphous PEI is a high-performance thermoplastic polymer frequently used for structural applications due to its remarkable mechanical performances and thermal stability, which are attributable to the aromatic imide groups in its molecular structure. Furthermore, PEI shows low smoke emission, flame retardancy, and chemical resistance properties, justifying its extensive use in aerospace and transportation applications. PEI is also employed as a feedstock material for FDM processes; nevertheless, the intrinsic porosity associated with AM techniques induces detrimental effects on polymer mechanical properties. To address this issue, PEI can be reinforced with nano- or micro-fillers such as carbon nanotubes (CNTs), graphene nano-platelets (GNPs), or carbon fibers (CFs) [112]. Karabel et al. [113] loaded PEI with carbon black (CB) to manufacture filaments for 3D printing, and they demonstrated that a low filler content (5 wt%) induces a 7% increase in the ultimate tensile strength in printed samples, comparable to the unmodified PEI obtained by traditional manufacturing processes. Moreover, FDM nanocomposites showed a 14% reduction in creep strain at 30 °C and a 19% reduction at 150 °C, thus enhancing the creep resistance of the matrix (Figure 21a). In this work, the CBs are preferred to other carbon-based fillers due to their cost-effectiveness, mainly. Whenever higher electrical conductivity is required, CNTs are necessary, as reported by Kaynan et al. [114]. In this work, PEI was loaded with different concentrations of CNTs (2 wt%, 5 wt%, and 7 wt%), and then processed by melt compounding to obtain a uniform diameter of filaments ready for 3D printing. Morphological analysis revealed that during processing, CNT undergoes breaking but despite this, an increase in the elastic modulus (up to 55%) was observed due to the efficient interfacial strength, dispersion, and CNT alignment in the PEI matrix. The surface conductivity of PEI was markedly improved by CNT incorporation, reaching a value of 2.57 × 10^−1^ S/cm. Chen et al. [115] dispersed CNTs in a PEI matrix to reduce the anisotropy of the printed manufacts, and the results show a slight increase in the tensile strength and modulus for 0° and ±45° raster angle printed samples; furthermore, for a 90° printing direction, the improvement was remarkably larger, up to 111.8% for the tensile strength and 89.5% for modulus, respectively, with addition of 1 wt% of CNT. Within the same work, post-production annealing treatments highlighted the further improvement in mechanical properties and a reduction in the warping of 3D-printed samples (Figure 21b); specifically, the annealing process should enlarge the interface and increase the degree of molecular entanglement between adjacent raster fibers [116].

### 3.7. Polyamide (PA)

Polyamide (PA) is one of the most important classes of synthetic thermoplastic polymers for engineering applications, primarily due to its exceptional comprehensive and tribological properties (nylon, as an example PA, is commonly employed to produce gears). However, FDM PA manufacts experience warping, distortion, and a lack of shape stability associated with the accumulation of thermal stress generated by crystallization of the polymer; this issue considerably narrows the fields of application of FDM-printed PA. Despite this, numerous works incorporate different fillers in PA as a strategy to reduce polymer crystallinity or hinder shrinkage level [117]. Rahim et al. [118] developed polyamide 12 (PA12) composites loaded with zirconia (ZrO_2_) and HA particles for FDM processes. Although the results report a reduction in the toughness and flexibility of the original polymer, the incorporation of inorganic fillers was found to improve the strength, stiffness, and thermal stability. The filler effect is twofold, i.e., it reduces both the coefficient of thermal expansion and the crystallinity of the matrix, limiting the aforementioned unwanted distorting effects. Zhu et al. [119] prepared PA12/GNP nanocomposites by melt compounding, aiming for an increase in the thermal conductivity of the printed system. Different filler contents were employed (2 wt%, 4 wt%, and 6 wt%), but the optimal results were obtained with lowest concentration, as for higher concentrations, particles aggregation occurred. Printed samples with a 0° raster angle report an increase in thermal conductivity and elastic modulus in the printing direction, respectively, of 51.4% and 7% compared to compression molding (CM). Moreover, ultimate strength does not undergo any detrimental effects due to the filler’s presence, maintaining the neat PA12 value. According to SEM analysis, the properties of FDM samples are likely higher than of CM samples due to the induced GNP orientation along the printing orientation during extrusion.

## 4. Properties and Applications of FDM Fiber-Reinforced Polymer Composites

The primary objective of the continuous fiber FDM 3D printing technique is to enhance the structural characteristics of the final element comprising a high-performing reinforcement and polymer matrix. Literature data indicate that tensile and flexural properties have received the majority of research efforts in the last decade; however, other properties, such as compressive strength, shear strength, impact strength, Young’s modulus, and fracture toughness still present potential for development [120].

Most of the research on 3D printing has used commercial fiber-reinforced filaments produced by commercial manufacturers, such as Markforged, MakerBot, Anisoprint, or Lulzbot TAZ [33], due to their superior control on fiber composition and geometrical tolerance. Despite the better fabrication accuracy, different researchers have developed their own FDM CFRC to take full advantage of designed customization in terms of matrix and fiber typology, reinforcement architecture, fiber length and volume fraction, and also sizing agents. The most commonly used polymers to produce FDM CFRP filament are PLA, ABS, PA6, PA, and PEEK [121]. It is well established that in continuous fiber-reinforced composites, mechanical properties are enhanced due to the load transfer between matrix and fiber. The same mechanism occurs in FDM CFRCs, as demonstrated by Matsuzaki et al. [122], who developed CF/PLA composites (inside the nozzle printing method) characterized by a 600% increase in tensile strength, compared to the neat PLA. According to Brenken et al. [123], the properties of FDM CFRC are comparable to those of aluminum-graded material if opportunely processed.

**Figure 22:**
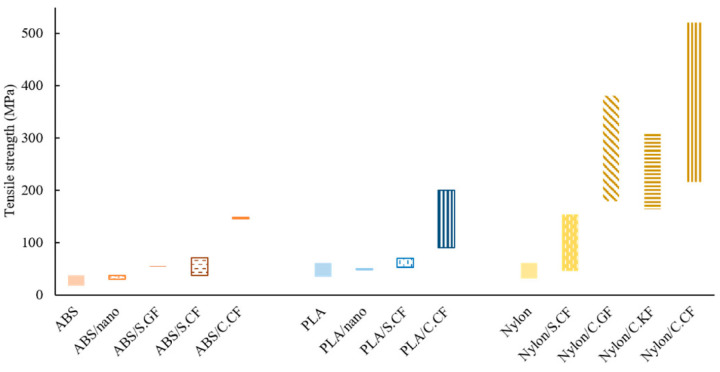
Tensile strength of FDM polymers (ABS, PLA, and nylon) loaded with different reinforcements—carbon fiber (CF), glass fiber (GF), Kevlar fiber (KF)—and architectures—nano-, short, and continuous [128].

Polyamine, in particular nylon, is among the polymers which can exhibit the most significant enhancement in terms of mechanical properties when reinforced with continuous carbon fibers, as demonstrated by Zhou et al. [124] who observed an eleven-fold increase in flexural strength for CF/nylon (395 MPa) compared to the neat matrix (32 MPa). Li et al. [125] strengthened PLA with CF (using an inside nozzle printing method), obtaining an improvement in tensile and flexural strength of +13.8% and +164%, respectively. Additionally, Yang [126] reinforced ABS with 10 wt% of CF, achieving a flexural strength of 127 MPa and a tensile strength of 147 MPa, which are comparable with traditional long-fiber composites. Liao et al. [127] developed FDM CFRC, considering different continuous CF contents in polyamide 12 (PA12) matrix. Their results indicated optimal performance at 10% fiber content, with a 2-fold increase in tensile strength and a 4-fold increase in flexural strength. Figure 22 reports tensile strength improvements for three different FDM polymers (i.e., ABS, PLA, and nylon) loaded with different fiber reinforcement types (i.e., nanofibers, short fibers, and CF). For all examined polymers, CFs present the highest efficiency to improve tensile strength and, in particular, the most relevant enhancement was reported for CF/nylon composites, reaching up to ten times the strength of the pure nylon.

**Figure 23:**
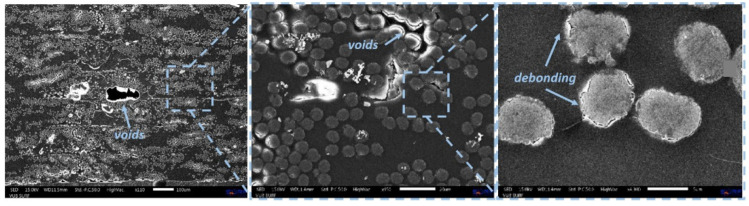
Micrographs of CF/PA composite section [129].

In analogy with the FDM printing technique of polymers and nanocomposites, and also in the case of CFRC FDM printing, setting the parameters plays a critical role. Raster angle, infill density, printing temperature, and speed are just some of the parameters that significantly affect the final performances of FDM manufacts. Pyl et al. [129] studied the effect of the raster angle on the tensile properties of CF/PA FDM composites, investigating four different configurations, i.e., [0°]_8_, [0/90°]_4s_, [0/90/±45°]_2s_ (quasi-isotropic), and [±45°]_4s_. Optimal results were recorded for unidirectional samples, with the tensile strength, strain to failure, and modulus of elasticity measured equal to 719 ± 46 MPa, 1.26 ± 0.09%, and 58.07 ± 1.86 GPa, respectively. Conversely, despite the lower tensile strength and modulus of the [±45°]_4s_ specimen, the strain to failure was found to be approximately four times higher than other configurations. It is noteworthy that these measured values are remarkably lower than the corresponding values found in traditionally manufactured composites, likely due to features like inhomogeneous fiber distribution, void content, and fiber debonding, as well as to low-volume fraction (only 27% compared to the 60% hold in traditional processes), as clearly assessed by the microscopy analysis of the final printed material (Figure 23).

Using a custom printing head, Heidari-Rarani et al. [130] succeeded in printing continuous carbon fiber-reinforced PLA matrix. The developed equipment consists of an inside nozzle head modified with a cooling fan capable of cooling down the printed filament quickly upon deposition, and this was found to improve the adhesion among raster fibers. Printed samples were investigated in terms of tensile and bending properties against the effect of printing temperature and speed, fiber tension, and fiber surface conditions. The experimental results indicate that these material properties rose up to 207% and 376%, respectively, of the pure PLA level for a fiber volume fraction of about 28.2%. Similar volume fraction levels were attained by Tian et al. [131] in CF/PLA composites, characterized by a flexural strength and bending modulus of 335 MPa and 30 GPa, respectively.

Nor et al. [132] implemented the Taguchi method, signal-to-noise ratio (S/N) approach, and analysis of variance (ANOVA) to study the effects of the layer thickness and the raster angle on the tensile strength of ABS and CF/ABS composites processed by FDM. It was found that layer thickness is the main parameter influencing the tensile strength of ABS printed parts, with a contribution of 86.95%, while the raster angle has a negligible effect; conversely, in CF/ABS composites, the raster angle contributes 75.14% to the tensile strength. The optimization of printing parameters by implementing such techniques can be achieved with a remarkably reduced number of experiments. The same ANOVA method was employed by Beylergil et al. [133] to optimize the Charpy impact strength of FDM-printed CF/PA composites. According to the attained results, only three factors have statistical relevance on this property: infill density, raster angle, and extrusion temperature. Data from the same work revealed that at a fixed CF weight content (i.e., 20 wt%), the highest impact strength of 10.54 kJ/m^2^ is achievable with an overall 150% efficiency compared to the lowest obtained result (i.e., 4.23 KJ/m^2^).

**Figure 24:**
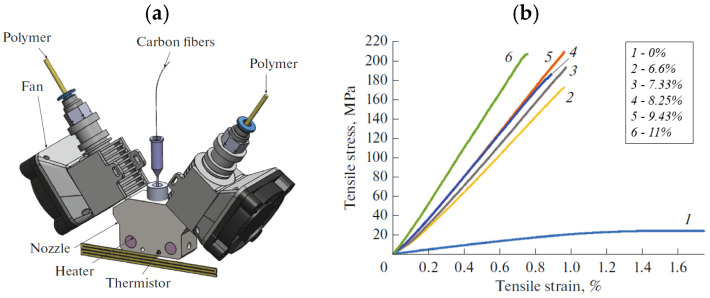
(**a**) Designed three-way wire-fed printing head; (**b**) tensile tests performed on ABS/CF at different continuous fibers content [135].

Matsuzaki et al. [122] highlighted that the inside nozzle printing method likely limits fiber impregnation, inducing a low interlayer shear strength and poor interfacial properties. Nakagawa et al. [134] developed a novel printing method comprising three main stages: firstly, ABS layers are printed on the printing bed by FDM process; then, bundled fibers, cut according to the specimen dimensions, are deposited and bonded to the printed layers by heating pins; ultimately, the upper layers of pure ABS are overprinted onto the reinforcing fiber layer. The resulting configuration of continuous CF sandwiched between the 3D-printed ABS layers revealed +1358% and +254% increases in tensile modulus and strength, respectively, if compared to neat ABS samples. Nevertheless, the improvement in mechanical strength is inherently related to the high CF content which, in turn, promotes the formation of air voids and a weak adhesion among fiber/ABS layers. To increase the interfacial performance, allowing for a smooth efficient load transfer mechanism, Prusinowski et al. [135] implemented a three-way wire-fed printing head enabling continuous CF-reinforced ABS printing. As shown in Figure 24, the reinforcing carbon fiber tow is inserted at the center of the head nozzle, between two separated channels, which supply the neat matrix filament into the printing nozzle element. The effects of the fiber content within the range from 6.6 to 11% and of the average print layer thickness from 300 to 500 μm were investigated to optimize the mechanical tensile strengths of the printed composites. The conclusions were that a maximum tensile strength (210.15 MPa) is achieved with 8.25 vol% fiber content and a 400 μm layer thickness. The poor fiber/matrix adhesion and the porosity within the FDM CFRC are the main identified driving mechanisms of failure. For composites manufactured by traditional techniques, it is well known that the expected failure mechanism is fiber breakage when the load is acting along the fiber direction due to its complete transfer to the reinforcement. More complex is the scenario of longitudinal loading configuration for FDM CFRC, as poor fiber/matrix adhesion and porosities promote fiber pull-out events, inducing a mixed-mode failure process (fiber breakage and fiber pull-out) [126]. The optimization of FDM process parameters with additional fibers’ treatment could surely improve fiber/matrix adhesion and enhance the final printed composite in terms of mechanical properties.

PEEK-based CFRCs are optimal substitutes for aerospace applications due to their excellent mechanical performances and outstanding physical properties, such as resistance to external radiation, exceptional thermal resistance, and stability. Nevertheless, the use of PEEK as a matrix in FDM CFRCs remains challenging due to its high melting point and melt viscosity, which hinder the reinforcing fibers’ impregnation, detrimentally affecting interlaminar (among layers) and intralaminar (fiber/matrix) adhesion. Meng et al. [136] used an assisted in situ laser to preheat an area on a printed layer before applying the next layer when printing CF-reinforced PEEK composites. The laser heats the area to a temperature above the PEEK glass transition temperature, improving the interlayer bonding. As a result, interlaminar shear strength (ILSS) and the flexural strength can reach over 35 MPa and 480 MPa, respectively.

One of the main advantages of thermoplastic composites is their recyclability. Tian et al. [137] developed a recyclable CF-reinforced PLA composite implementing a hot air-gun technique which allows for the pull-out of carbon fibers from the PLA matrix by heating the composite above the melting temperature. This technique enables the very efficient recovery of both PLA and carbon reinforcement (73% and 100%, respectively). The secondary CF/PLA printed materials are characterized by a 25% improvement in flexural strength in comparison with the original 3D-printed composites, likely due to further PLA impregnation of the CF during the recycling and melting processes, which enhances the CF/PLA interface strength.

## 5. Conclusions

This review summarizes the recent progress in thermoplastic composites used in FDM processes. The review covers particles/nanoparticles, short fibers, and long fibers as reinforcing agents, and identifies the critical parameters of this advanced technique to produce innovative feedstock filaments, along with their corresponding performance.

The effects of different fillers on ABS, PLA, PVA, TPU, PEI, PA, and PEEK polymer matrices have been analyzed, alongside discussion of the advantages and limitations of each material and their impact for specific applications.

Finally, the review discusses FDM fiber-reinforced polymer composites, highlighting the three methods used to incorporate continuous fibers into a matrix: “prior to nozzle”, “inside the nozzle”, and “after the nozzle”, summarizing recent data on the mechanical properties.

Despite recent progress and advancements, this research area still presents challenges and opportunities for future work to focus on the enhancement of filler dispersion and interface bonding; improvement of the printability of highly loaded composites; enhancement of the mechanical properties of FDM-printed parts; the addition of specific functionality properties in the FDM feeding materials (thermal stability, minimum shrinkage, coefficient of thermal expansion, thermal or electrical conductivity); and the development of sustainable and environmentally friendly FDM feedstocks.

Indeed, addressing and advancing these challenges will lead to high-performance, functional, and sustainable materials for a wide range of applications in different industrial sectors.

## Figures and Tables

**Table 2:** FDM ABS-based composite properties.

Matrix	Filler (Content)	Δσ^T^_fil_ [%]	ΔE_fil_ [%]	Δσ^T^ [%] (PD–PO)	ΔE [%] (PD–PO)	Δσ^f flex^ [%] (PD–PO)	ΔE ^flex^ [%] (PD–PO)	ρ_fil_ [Ωcm]	ρ_3D_ [Ωcm] (PD–PO)	REF
ABS	MWCNT (2 wt%)	-	-	+41.8 (±45–F)	-	-	-	-	-	[58]
ABS	MWCNT (6 wt%)	+10.0	+18.9	+54.2 (C–F) +12.4 (±45–F) −15.0 (C–UR)	+22.3 (C–F) +18.7 (±45–F) +5.0 (C–UR)	-	-	4	62 (C–F) 50 (±45–F) 15 (C–UR)	[59]
ABS/LLDPE	Short glass fibers (5 wt%)	-	-	+139.1 (0–F)	-	-	-	-	-	[60]
ABS	Oil palm fiber (3 wt%)	-	-	+6.9 (0/90–F)	+4.5 (0/90–F)	−51.7 (0/90–F)	-	-	-	[61]
ABS	Jute fiber (5 wt%)	-	-	−9 (0/90–F)	+0.9 (0/90–F)	+42.9 (0/90–F)	-	-	-	[62]
ABS	Nano-montmorillonite (1 wt%)	-	-	+25.8 (0–F) +53.0 (0–UR)	-	+17.1 (0–F)	+21.2 (0–F)	-	-	[64]
ABS	CNT (3 wt%)	+10.0	+10.0	−34.8 (C–UR) +13.5 (±45–F) +11.0 (C–F)	-	-	-	10^2^	10^7^ (C–UR) 10^9^ (±45–F) 10^9^ (C–F)	[66]
ABS	MWCNT (7 wt%)	-	-	+29.2 (C–F) +12.5 (±45–F)	+47.5 (C–F) +27.4 (±45–F)	-	-	-	10^4^ (C–F) 10^3^ (±45–F)	[67]
ABS	Perlite microsphere (20%)	-	-	−12.1 (±45–F)	+17.3 (±45–F)	-	-	-	-	[68]
ABS	Cellulose nano crystal (2 wt%)	+2.6	+52.5	-	-	-	-	-	-	[69]

The footnote “fil” refers to the value evaluated on the filament; PD = printing direction; PO = printing orientation; C = concentric PD; F = flat PO; UR = upright PO.

**Table 3:** FDM PLA-based composite properties.

Matrix	Filler (Content)	Δσ^T^ [%] (PD–PO)	ΔE [%] (PD–PO)	Δε [%] (PD–PO)	Δσ^f flex^ [%] (PD–PO)	ρ_fil_ [Ωcm]	ρ_3D_ [Ωcm] (PD–PO)	REF
PLA	GNP/MWCNT (1.5/4.5)	-	-	-	-	-	0.17 (0/90–F)	[71]
PLA	Hemp (5 wt%)	-	+65 (±45–F)	-	-	-	-	[70]
PLA	Al (7 wt%)	−16.6 (0–F)	−1.7 (0–F)	+23.6 (0–F)	-	-	-	[74]
PLA	SiC (5 wt%)	+56.5 (±45–F)	+31.4 (±45–F)	-	-	-	-	[77]
PLA	Graphite (1 wt%)	+41.0 (0/90–F)	-	-	-	-	-	[78]
PLA	GNP (10 wt%)	+29.0 (±45–F)	+34.0 (±45–F)	−11.0 (±45–F)	-	20	102 (±45–F)	[79]
PLA	CNT (6 wt%)	+64.1(0–F)	-	-	29.3 (0–F)	-	10^3^ (0–F)	[82]
PLA	Biochar (3 wt%)	+89 (Triangle–F)	+100 (Triangle–F)	+45 (Triangle–F)	-	-	-	[83]
